# Resting-state networks in the course of aging—differential insights from studies across the lifespan vs. amongst the old

**DOI:** 10.1007/s00424-021-02520-7

**Published:** 2021-02-12

**Authors:** C. Jockwitz, S. Caspers

**Affiliations:** 1 Institute of Neuroscience and Medicine (INM-1), Research Centre Jülich, Jülich, Germany; 2 Institute for Anatomy I, Medical Faculty & University Hospital Düsseldorf, Heinrich Heine University, Düsseldorf, Germany; 3 JARA-Brain, Jülich-Aachen Research Alliance, Jülich, Germany

**Keywords:** Resting state, Functional connectivity, Aging, Lifespan, Older adults

## Abstract

Resting-state functional connectivity (RSFC) has widely been used to examine reorganization of functional brain networks during normal aging. The extraction of generalizable age trends, however, is hampered by differences in methodological approaches, study designs and sample characteristics. Distinct age ranges of study samples thereby represent an important aspect between studies especially due to the increase in inter-individual variability over the lifespan. The current review focuses on comparing age-related differences in RSFC in the course of the whole adult lifespan versus later decades of life. We summarize and compare studies assessing age-related differences in within- and between-network RSFC of major resting-state brain networks. Differential effects of the factor age on resting-state networks can be identified when comparing studies focusing on younger versus older adults with studies investigating effects within the older adult population. These differential effects pertain to higher order and primary processing resting-state networks to a varying extent. Especially during later decades of life, other factors beyond age might come into play to understand the high inter-individual variability in RSFC.

## Introduction

Population aging inherits a shift in the age distribution of our population that has a tremendous impact on health and economic aspects of our society, as it is particularly accompanied by increasing prevalences of neurodegenerative diseases, such as Alzheimer’s disease [29]. Normal, non-diseased aging is already associated with considerable alterations to the brain and its associated functions. Cognitive functions decline, most prominently in the domains of attention, executive functions and memory [30, 31], prevailing current research to focus on the relationship between brain structure, brain function and cognitive performances [55, 64, 65, 90]. Based on mainly cross-sectional studies, relations between increasing brain atrophy and cognitive performance decline during the aging process could be demonstrated ([63, 69]; for a recent review on longitudinal changes, see [54]). Based on task-based functional magnetic resonance imaging (fMRI), theories about a functional reorganization of the brain during the aging process were proposed (e.g. [11, 15, 24, 65]).

As task-based fMRI is typically limited in terms of general coverage of functional networks across the entire brain, assessment of resting-state functional connectivity (RSFC) for a more systemic investigation of the functional brain architecture came into focus over the last decade, particularly also in view of age-related changes in RSFC. RSFC relies on spontaneous fluctuations of the blood-oxygenation level dependent (BOLD) signal (0.1–1.0 Hz) in the brain during rest; i.e. participants are usually lying in the MR scanner, without performing any task [6]. Spatially distinct regions that are supposed to work together during cognitive tasks exhibit similar spontaneous fluctuations and are thus functionally linked [6, 7]. For example, primary processing networks represent those functional networks involved in somatomotor functions (somatomotor network), auditory processing (auditory network) or visual processing (visual network). Higher order functional networks represent those involved in higher cognitive functions, such as introspection (default mode network), executive functions and attention (executive network), language processing and working memory functions (frontoparietal networks) or memory performance and emotional processing (limbic network) [42, 75]. Changes to these resting-state networks, e.g. due to aging, are supposed to reflect more fundamental alterations or adaptations at the general level of brain function. For example, changes in RSFC might represent disturbed communication of the anatomical connectivity (i.e. due to disruptions in white matter tracts) and have been associated with neurotransmitter dysfunction (i.e. dopamine), less glucose metabolism and resulting increases in amyloid binding [18, 73]. So far, investigations of RSFC differences due to aging are highly variable between studies. Differences in preprocessing (for recent reviews and studies on e.g. motion correction and global signal regression, see [21, 27, 88]), brain regions or networks investigated, inclusion of covariates of (non-) interest, sample characteristics and approaches used, however, hamper generalizability of the so far obtained results. For example, hypothesis-driven approaches focus on either the functional connectivity between two brain regions (seed-to-seed) or between an initial region of interest and all other voxels of the brain (seed-to-whole brain). Data-driven approaches, e.g. independent component analysis (ICA), decompose the spontaneous fluctuations of the BOLD signal into a set of independent components [3, 4] that represent resting-state networks, such as the default mode network (DMN; [26, 62]), the visual network, somatomotor network, auditory network, left and right frontoparietal networks, executive network, limbic network and salience network (e.g. see [75, 76]). Finally, graph-theoretical approaches are probably the most integrative way to investigate RSFC. Based on predefined parcellations (which might be theory or data driven , e.g. see [59, 70, 93]), graph-theoretical approaches assess RSFC at both node and system level and allow for investigation of function brain network segregation (i.e. within-network RSFC) as well as integration (i.e. between-network RSFC) [76, 78].

RSFC has been used in various ways to answer the question of how the functional architecture of the brain ages. Focusing on the older adult population in particular reveals an additional sample characteristic which might explain parts of this heterogeneity: inter-individual variability increases considerably over the lifespan, at this characterizing the old age. This introduces a new variable when studying typical effects in the aging brain which might be obscured when studying lifetime trajectories and only seizable when particularly focusing on the older adult population. The current review aims at summarising and discussing so far obtained results from the most prominent papers with respect to age-related differences in RSFC with a specific focus on the challenge of studying lifetime trajectories vs. focusing on specific age ranges and associated sample characteristics.

## Age-related differences in RSFC: a matter of sample characteristics?

There is a vast amount of literature investigating age-related differences (cross-sectional) or changes (longitudinal) in terms of RSFC. The following two sections review studies that investigate differences/changes in RSFC from young to late adulthood and within the older adult population, respectively.

## Age-related differences in RSFC across the lifespan

The resting-state network probably studied most intensively with regard to aging effects is the DMN. Functional connectivity within the DMN has repeatedly been shown to decrease from early to late adulthood. This has been studied using seed-based approaches, graph theory as well as ICA. Participants’ age ranges from young to older ages across the whole adult lifespan, while other studies compared younger versus older adult groups. These different approaches nevertheless came to the same conclusions, as exemplified here on three different studies investigating the effects of aging within the DMN: Andrews-Hanna et al. [1] compared 38 younger (mean age = 22 years) and 55 older adults (mean age = 77 years) using a seed-based approach, Geerligs et al. [22] used a graph-theoretical approach and compared 40 young (mean age = 21 years) to 40 older adults (mean age = 65 years), and Mowinckel et al. [47] used an ICA in a larger group of 238 adults ranging from 21 to 81 years of age. Based on these analyses, which differ in many aspects, i.e. age regression versus group comparison, theory- versus data-driven, there seems to be a general consensus that the DMN shows decreases in RSFC from early to late adulthood (see Table 1 for more studies showing similar age-related decreases in RSFC of the DMN).

**Table 1:** Overview of cross-sectional studies assessing age-related differences within major resting-state networks

Study	Method	Age group	*n*	DMN	Higher order					Primary processing			Others
					DAN	VAN/SN	FPN/CN	LN	EXE	SMN	AN	VN	
Andrews-Hanna et al. [1]	Seed	Y (22.4 ± 3.6) O (76.5 ± 8.2)	38 55	↓	↓								
Geerligs et al. [22]	Graph theory	Y (20.6) O (64.9)	40 40	↓			↓			**=**		**=**	CO ↓
Song et al. [77]	Graph theory	Y (24.6 ± 3.3) O (58.0= ± 6.1)	26 24	↓	**=**	**=**	**=**			↑			CO & Subc &Cereb =
Zhang et al. [94]	Seed	Y (23.96 ± 1.8) O (69.86 ± 5.8)	18 22	↓	↓	↓			↓			**=**	
Damoiseaux et al. [14]	ICA	Y (22.8 ± 2.3) O (70.7 ± 6.0)	10 22	*A ↓* *P =*									
Grady et al. [25]	Graph theory	Y (22.4 ± 3.1) O (69 ± 5.2)	45 39	↓	↓		**=**						
Spreng et al. [79]	Seed	Y (24 ± 3.8) O (74.6 ± 3.8)	54 61	↓	↓								
Betzel et al. [5]	Graph theory	7–85 years	126	↓	**=**	↓	↓	**=**		**=**		med = lat ↓	
Tomasi et al. [85]	FC density	13–85 years	913	↓	↓					↑			Cereb ↑
Varangis et al. [87]	Graph theory	20–80 years	427	**=**	↓	**=**	**=**			mouth ↓ hand =	↓	**=**	CO ↓
Chan et al. [13]	Graph theory	20–89 years	210	↓	**=**	VAN*↓* SN=	↓			**=**	↓	↓	CO ↓
Mowinckel et al. [47]	ICA	21–81 years	238	↓			L* ↑* R* ↑*		↑	↑	↑	med ↓ lat ↓	
Onoda et al., [53]	Seed	36–86 years	73	↓		↓	=			=		↓	Cereb & Temp =
Siman-Tov et al. [74]	Graph theory	Y (25.5 ± 4.8) M (50.6 ± 5.4) O (69.0 ± 6.3)	543 238 106	YM ↓ MO =	YM ↓ MO =	YM ↓ MO =	YM ↓ MO =			YM ↑ MO ↓	YM ↓ MO ↓	YM ↓ MO ↓	
Huang et al. [34]	ICA	51–85 years	430	V *↓* A & P *=*			L = R *↓*		**=**	↓	↓	med ↓ / lat =	Cereb=
Stumme et al. [82]	Graph theory	55–85 years	772	**=**	**=**	**=**	**=**	**=**	**=**	↓		↓	
Jockwitz et al. [39]	ICA	55–85 years	711	**=**			*L ↑* *R ↑*		↑				
Zonneveld et al. [95]	ICA	50–95 years	2878	A* ↓* P* =*	**=**	↓	**=**			↓		↑	Subc & Temp =

*↓* RSFC decrease, *↑* RSFC increase, *=* RSFC stability, DMN = Default Mode Network, DAN = Dorsal Attention Network, VAN = Ventral Attention Network, SN = Salience Network, FPN = Frontoparietal Network, LN = Limbic Network, EXE = Executive Network, SMN = Somatomotor Network, AN = Auditory Network, VN = Visual Network, CO Cingulo-Opercular Network, Subc = Subcortical, Temp = Temporal, Cereb = Cerebellum, Y = Young, M = Middle, O = Old, A = Anterior, P = posterior, V = ventral, med = medial, lat = lateral, L = left, R = right

For other functional networks, however, results are more multifaceted when it comes to aging. Mowinckel et al. [47] showed age-related decreases for the visual network, as well as increases in RSFC for the somatomotor, auditory, executive and frontoparietal networks from early to late adulthood. In contrast, using a graph-theoretical approach, Betzel et al. [5] found within-network decreases in RSFC for higher order control and attention networks, as well as stable primary processing visual and somatomotor networks in a group of 126 participants between 7 and 85 years of age. Using RSFC local density mapping, Tomasi and Volkow [85] showed decreases for the dorsal attention network, but increases for the somatomotor and cerebellar networks in a large sample of 913 participants ranging from 13 to 85 years of age. While the aforementioned studies mainly used large age ranges (i.e. from teenage to old age), Siman-Tov et al. [74] conducted a multicentre study and divided their large sample into 543 young (21–40 years), 238 middle-aged (41– 60 years) and 106 older adults (≥ 61 years). Interestingly, they showed a twofold effect of age on RSFC: the salience, dorsal attention and frontoparietal networks seem to decrease in within-network RSFC from young to middle age, with less decreases (or even slight increases) in RSFC from middle to older age. In turn, the auditory and visual networks showed linear decreases from early to middle to late adulthood, while the motor network rather showed non-linear trajectories during the lifespan, i.e. increases in RSFC from young to middle adulthood with a subsequent decrease in RSFC from middle to late adulthood. For an overview of within-network RSFC differences with increasing age, see Table 1. What becomes obvious when summarizing the results obtained so far regarding particularly within-network functional connectivity in the course of age is that predominantly higher order networks show RSFC decreases with increasing age. Primary processing networks on the other hand, i.e., somatomotor, auditory and visual networks, show more heterogeneous results when it comes to aging from early to late adulthood, with some studies showing increases and others reporting stability or decreases in RSFC with increasing age. Referring back to Siman-Tov et al. [74], who subdivided their sample into young, middle-aged and old adults, a non-linear relationship between age and RSFC at least within the somatomotor network might explain the heterogeneity of the results best. Generally, age-related within-network RSFC decreases have been associated with arespective decline in mental functions [1, 67, 72, 86].

As the within-network RSFC focuses on single functional networks, another relevant aspect for understanding the functional architecture of the aging brain concerns the between-network RSFC, i.e. the question of integrated processing across networks. Overall, age-related increases in RSFC between networks were found, such as for the dorsal attention network, the salience and ventral attention networks and the somatomotor network [5], between subcortical and higher order networks [23] or between the control network and the visual network as well as DMN [22]. Likewise, research groups directly investigate the relationship of within- versus between-network RSFC by examining the ratio between the two. With increasing age, functional brain networks tend to be less segregated, resulting from a combination of between-network RSFC increases and/or within-network RSFC decreases [13, 25]. At younger age, functional brain networks are typically more segregated, with every network being relatively specialized for distinct mental processes [13]. These functionally specialized brain networks interact, at least to a certain degree, with each other, to fulfil everyday task requirements. With increasing age, however, the functional coherence within these networks decreases, while the between-network RSFC increases. This would hint at functional dedifferentiation during aging, i.e. a loss of functional specialisation of specific brain networks as the brain ages [24]. This would be also supported by studies investigating the relation between resting state and task-based functional connectivity; e.g. Hughes et al. [35] found that functional connectivity across the two states were less correlated in older compared to younger subjects. The authors explained this effect by a lower segregation (i.e. specialisation) of functional brain networks, which then potentially might lead to disturbed communication of brain networks during task performance. As suggested by established aging theories, though, the brain reorganizes itself to maintain cognitive functions as stable as possible. Thus, the observed increase of integration across brain networks might not only be a consequence of functional dedifferentiation processes, expressed by a loss of within-network RSFC in the course of aging. Rather, increases in between-network communication might reflect compensational mechanisms to maintain cognitive functions as stable as possible (e.g. scaffolding theory of aging [65], hemispheric asymmetry reduction in older adults [11] or posterior-to-anterior shift in aging [15]).

Taken together, lifespan trajectories of RSFC are characterized by reductions of functional coherence within brain networks, together with enhanced RSFC between functional brain networks, which has been shown for the DMN as well as higher order brain networks. Primary processing networks (i.e. somatomotor networks) on the other hand are potentially subject to non-linear changes throughout the lifespan, which cannot be uncovered by exploring linear changes from early to late adulthood. Consequently, investigations of the older adult population could help to reveal age-related changes in the functional brain architecture.

## Differences in RSFC within the older adult population

The so far reported studies describe age-related differences in RSFC from early to late adulthood. With an increasing number of older subjects in our societies and the burden of increasing prevalence of neurodegenerative diseases, though, the need for understanding the particularities of the old brain is of specific relevance. Two observations from lifespan studies support this notion: the hints on non-linear aging trajectories [74] and the increasing inter-individual variability over the aging process, with regard to the brain functional network architecture [1, 47], brain structure [16] and cognitive performance [52], with a possible starting point in the mid-50s [31, 47]. Investigating a population-based cohort of older adults (between 55 and 85 years of age) using ICA, Jockwitz et al. [38] found again high inter-individual variability of RSFC in all major brain networks. This was especially seen for the DMN, together with stable RSFC in this group of older adults. This finding was corroborated in another study on the same cohort but using a graph-theoretical approach [82], as well as by investigation of other cohorts and by using seed-based analyses (age ranges: 64–85 years [33]; 69–85 years [74]). Other studies, contrarily, showed further decreases in RSFC for the DMN during older ages (64–91 years [40]; 50–95 [95]), but only for the anterior part of the DMN, while the posterior DMN showed increases or remained stable during older ages. Additionally, Huang et al. [34] could show that only a ventral DMN (representing a further subdivision of the posterior DMN described by other authors; comprised of the inferior parietal lobule and parts of the posterior cingulate cortex) revealed a decrease in RSFC in 430 adults being 51 years and older. Dividing the older adult population into age groups, Farras-Permanyer et al. [17] showed a slight decrease within the anterior and ventral DMN from 60 to 79, with a subsequent increase in subjects being older than 80 years of age.

In light of the lifespan studies discussed in the first part of this review, the current state of research clearly indicates that functional connectivity within the DMN from early to late adulthood decreases as a function of age. During older ages, however, these whole network differences in RSFC cannot be extracted. Instead, investigating only parts of the DMN in this population revealed decreases in functional connectivity amongst the older subjects, hinting at more subtle differences at older ages. The question that thus remains is: If age itself does not explain the observed inter-individual variability, which other factors could be accountable for that?

With respect to age-related differences in higher order networks within the older adult population, quite heterogeneous observations have been reported. While Jockwitz et al. [38] showed slight increases in RSFC for the frontoparietal and executive networks during older ages using ICA, Zonneveld et al. [95] showed a decrease in within-network RSFC for the ventral attention network, while the dorsal attention, temporal and frontoparietal networks remained stable.

Regarding the primary processing networks, Siman-Tov et al. [74], Stumme et al. [82] and Huang et al. [34] could show a decrease in RSFC particularly within primary processing networks, i.e. somatomotor and/or visual networks. Contrarily, Farras-Permanyer et al. [17] and Varangis et al. [87] found stable somatomotor and visual network RSFC amongst older subjects (65–80 years), and Zonneveld et al. [95] reported increased RSFC for the visual network together with decreases in RSFC for the somatomotor network. This observation is especially interesting since the primary processing networks already showed heterogeneous age-related differences when comparing younger and older adults, and the somatomotor network most likely follows a non-linear age trajectory from early to late adulthood. While from young to old age, the somatomotor network predominantly showed increases in RSFC (cf. Table 1), its within-network RSFC seems to rather decrease (or at least remain stable) within the group of older subjects.

Similar to the heterogeneity of within-network RSFC, between-network RSFC differences with age show a heterogeneous picture. Zonneveld et al. [95] showed both increases and decreases in RSFC between networks, i.e. increased RSFC between the DMN and higher order networks as well as between subcortical and temporal networks, together with decreases in RSFC between anterior and posterior DMN, ventral and dorsal attention networks as well as ventral attention network and somatomotor network. Contrarily, Stumme et al. [83] found increased connectivity between the somatomotor network and higher order networks, but RSFC decreases between the somatomotor and visual network. The higher inter-network communications have been interpreted as an attempt to compensate for the decline of primary processing networks to maintain mental abilities as stable as possible from early to late adulthood. A similar mechanism might also apply to the older adult population. Taking recent evidence about the reserve capacity of the brain [81] into account, though, the question remains if and to what extent such compensational mechanisms are actually present in the already aged brain. The heterogeneous results strongly emphasize the necessity for more research within the older adult population to extract clear hypotheses regarding functional reorganization within the older adult population and the relevance of the factor age in this regard.

## Other factors that might explain the high inter-individual variability in older adults

Comparing lifespan development of RSFC from early to late adulthood and changes in RSFC in the older adult population brings up an important aspect. The two groups are not directly comparable regarding age-related differences in RSFC extracted from cross-sectional data: From early to late adulthood, the DMN as well as higher order networks decreases in within-network RSFC, independent of methods (i.e., seed-based, ICA, graph theory) and statistical approaches (i.e., group comparisons, age-regression) used. During older ages, however, RSFC remains rather stable for those networks. Increasing inter-individual variability over the aging process might be one essential factor that explains these differences. From early to late adulthood, a large part of the inter-individual variability in RSFC is attributable to the factor age. During older ages, however, this variability across subjects has proven to be even higher, and age itself cannot fully account for this variance. Consequently, other factors might come into the fore that explain changes in RSFC during older ages.

The question of which factors might be essential in this regard, however, has not been answered so far. Recent studies focused on quite different factors, which can be assigned to modifiable and non-modifiable (risk) factors. With respect to the non-modifiable (risk) factors, Zonneveld et al. [95], for example, showed that sex might be important. Sex differences in RSFC of the aged brain have been reported repeatedly, such that females show higher connectivity within the DMN, while males show higher RSFC for the somatomotor network [66, 83, 95]. Recently, Weis et al. [91] even showed that RSFC serves as a useful basis for sex prediction. This pertains important particularly when taking sex differences in prevalences of neurodegenerative diseases into account. Another large and in itself also heterogeneous group of potentially relevant factors in the aging brain is the number of physical factors, such as body size, that have been reported to be related to both within- and between-network RSFC [46, 95]. Genetic variants have also been discussed over the last decade to explain aging-related alterations of the functional architecture of the brain. Especially, the APOE status, a common risk factor for Alzheimer’s disease, came into focus also in relation to normal, non-diseased aging (for a recent review, see [57]). Wu et al. [92] showed altered RSFC for the executive and salience network as well as the DMN in 17 APOE 4 carriers between 50 and 65 years of age, while Zonneveld et al. [95] did not find any APOE-associated effects in their large sample of 2878 older adults.

With respect to modifiable factors, Boraxbekk et al. [9] showed a higher RSFC within the posterior DMN with increasing physical activity. Pillemer et al. [58] found higher RSFC within the sensorimotor, visual, insular and frontoparietal networks with higher social interactions. Furthermore, Lindbergh et al. [43] found a stronger segregation of the DMN, expressed by a higher within- compared to between-network RSFC, over the course of aging to be associated with higher intelligence. Bittner et al. [8] integrated different aspects of lifestyle into a lifestyle risk score including smoking and alcohol intake as risk factors and social interaction and physical activity as protective factors. Higher lifestyle risk was associated with higher between-network RSFC, i.e. between motoric and dorsal occipital cortex. Thus, protective factors that are supposed to increase the so-called cognitive reserve capacity during aging [80, 81] are associated with a better perseveration of functional brain networks in older adults. Moreover, nutrients and dietary behaviour also showed associations with RSFC in the older adult population: Caloric restriction was associated with higher RSFC between the hippocampus and left precuneus and angular gyrus, together with lower RSFC between hippocampus and right inferior frontal gyrus [60]. Mega-3 polyunsaturated fatty acids were associated with higher RSFC within frontal lobe regions [84], and vitamin B1 and B6 were found to be associated with higher interhemispheric RSFC [37].

The integrated analysis and systematic examination of the large number of factors as a whole contributing to the high inter-individual variability of RSFC in the aging brain gains particular importance within the older adult population. Miller et al. [46] started to use such an approach by investigating a variety of lifestyle and physical factors in relation to RSFC in a population-based cohort consisting of more than 5000 older adults. It seems that although not each variable was significantly associated with RSFC, a large number of variables show at least small associations with RSFC. This shows that such systematic global investigations provide valuable hints at potentially relevant associations between RSFC and factors beyond age. Hence, the ultimate goal would be to reassemble these small but possibly important aspects and to disentangle the individual contributions to the aging process of the brain to finally get a clearer picture of age-related changes in the functional architecture of the brain.

## Longitudinal investigations into the aging process and relations to cognitive performance

So far, research investigating age trajectories of RSFC mostly focused on comparing subjects of different age, i.e. between-subject differences. While these differences in RSFC might be related to cohort effects, rather than reflecting a true aging effect [71], there are only limited studies investigating RSFC using a longitudinal research design (e.g. [44, 51]). However, to understand the aging process within subjects, it is necessary to explore intra-individual changes of the functional architecture and related reorganization of the aging brain. Important to note, changes seen in longitudinal studies are usually smaller and more focused to specific networks. For example, Malagurski et al. [44] found RSFC to decrease within the frontoparietal network over a time interval of 4 years. In turn, the limbic and somatomotor networks seem to increase with respect to the within-network RSFC. Additionally, increases in the between-network RSFC relating especially to the somatomotor network (e.g. with the frontoparietal, DMN, limbic, ventral attention and dorsal attention networks) have been reported. Ng et al. [51] showed the DMN and frontoparietal networks to decrease over a time interval of 2 years, while their between-network RSFC remained stable. Fjell et al. [19] showed several networks (i.e. frontoparietal and DMN) to increase in within-network RSFC over time. While longitudinal investigations of changes in RSFC are an inevitable step towards a better understanding of intra-individual trajectories of aging, more research is needed to reliably extracting robust trends.

One additional important issue concerning the assessment of age-related changes in RSFC is cognitive performance itself. Resting-state networks are supposed to be engaged in the successful performance of distinct cognitive functions. While networks such as the visual, auditory and somatomotor networks are supposed to be involved in primary processing of the respective function, higher order networks are associated with higher cognitive functions. The DMN, for example, seems to be involved in introspection, day dreaming and episodic memory collection; frontoparietal and executive networks are rather associated with attention, executive functions and working memory [42, 75]. During the aging process, when RSFC seems to change, this could be somehow related to cognitive abilities and corresponding decline. And indeed, several studies reported an association between RSFC and cognitive status. In cross-sectional studies, RSFC has frequently been associated with cognitive performance level [1, 20, 23, 38, 48, 49, 53, 83, 87]. However, cognitive tasks and brain metrics investigated differ between studies, making it difficult to compare them directly. For example, Andrews-Hanna et al. [1] showed that within-network RSFC of the DMN was positively related to memory functions. Perry et al. [56] further added that higher between-network RSFC (reflecting a lower segregation of functionally specialised brain networks) would be associated with worse overall cognitive function.

Regarding longitudinal analyses, several studies assessed associations between RSFC and cognitive performance by either focussing on level-change (i.e. the relation between a cross-sectional measured level of performance or RSFC and the longitudinal change of these metrics over time) or change-change (i.e. the relation between two longitudinal changes over time) associations [13, 20, 44, 51]. For instance, a decline in processing speed has been associated with increases in between-network RSFC of the executive network and DMN [51], and a decline in segregation of the frontoparietal network over time [20, 45] further showed stability in within-network RSFC over time to be related to better episodic memory performance. Thus, regarding the relation between RSFC and cognitive performance, cross-sectional and longitudinal studies indicate that higher segregation of specialized functional brain networks might be related to better cognitive performance. Nevertheless, especially more longitudinal analyses are warranted to better understand this relationship.

## Conclusions and future investigations

Research in the field of RSFC has become quite popular during the last decades, since it assesses the underlying functional architecture of the brain that is supposed to be responsible for successful cognitive performance and related to pathological conditions, e.g. Alzheimer’s disease. Based on the current state of research, general trends on age-related alterations of the resting-state functional network architecture of the brain can only partially be extracted, due to differences in analysis methods, investigated networks or sample characteristics across studies. Two main conclusions can be drawn from the current literature. First, the DMN seems to decrease from younger to older ages, but within the older adult population, the DMN as a whole, at least in normal aging, remains relatively stable, with only anterior parts (further) deteriorating. This is an important aspect since the DMN has been reported to be especially vulnerable to age-related changes. From the studies reported in the current review, we can conclude that this age-related vulnerability pertains to the whole adulthood, while during older ages, the variability between subjects hamper the extraction of major trends. Thus, age itself might be less important during older ages. Here, pathological states, e.g. Alzheimer’s disease, might be of special importance. Sheline and Raichle [73], for example, showed that the DMN is prone to preclinical changes in the network configuration, thus serving as a clinical target for early detection and therapy.

Second, especially the primary processing networks seem to exhibit heterogeneous and largely non-linear effects over the lifespan when generalizing over different study samples and methods used. Regarding the somatomotor network, age-related decreases in within-network RSFC during older ages might hint at a dedifferentiation of this network, especially pertaining to the older adult population. This is an important observation since it emphasizes differential aging during older ages that might be related to cognitive impairments. Especially with respect to the older adult population, it has to be mentioned that the studies described here included subjects of different age ranges (i.e. [38] included subjects from 55 years onwards, while Huang et al. [34] included subjects being 50 years and older), which might impact the obtained results. Nevertheless, the heterogeneity of results together with the observed inter-individual variability emphasizes the necessity of investigating other determinants beyond the factor age which might be relevant for explaining the heterogeneous findings in aged resting-state networks.

Large sample sizes are needed to have the sufficient power to even detect these sometimes subtle, but still important effects on the aged brain [10]. Recent studies already showed that other factors, such as lifestyle, genetics and physical factors, might influence the functional architecture in the aging brain. Furthermore, the here discussed metricsincluded within- and between-network connectivity. It has to be mentioned, though, that other metrics, e.g. participation coefficient, weighted degree or dynamic functional connectivity, are becoming more prominent to assess RSFC from quite different perspectives. Their relevance for understanding the aging process need to be further assessed.

In addition to this, although the resting-state fMRI has proven to be an essential tool to examine the underlying architecture of the brain, with respect to healthy as well as pathological conditions, it has to be mentioned that this technique, nevertheless, inherits some methodological as well as psychological challenges. First, fMRI is known to be an indirect measure of neuronal activity, with a slow time resolution, as it is induced by the slow hemodynamic response, compared to the much faster neuronal firing rate. Although multimodal analyses, e.g. combined fMRI and EEG, provide valuable information in this respect, the exact relationship between the two remains unclear. Furthermore, it has to be mentioned that the resting-state fMRI is related to several psychological states, e.g. anxiety arousal and uncomfortability (e.g. [32, 41, 50]), which in turn might be related to physiological conditions, e.g. motion, cardiac and respiratory responses. Although several cleanup tools try to remove confounding effects of motion, heartbeat or breathing (e.g. AROMA [61] and FIX [28, 68] implemented in FSL), the relation between psychological states and RSFC is not directly measurable and therefore remains within the signal. These effects might, of course, represent confounding factors during the aging process**.**

Furthermore, current studies mainly used a cross-sectional design, which prevents drawing conclusions about potentially causal relationships. So far, there are only limited studies investigating RSFC using a longitudinal research design (e.g. [44, 51]). With large population-based cohorts across the lifespan or with focus on the older age recently being available and currently being built, investigating subjects at more than one time point, including neuroimaging assessment together with a multitude of phenotypes and influencing factors (e.g. 1000BRAINS [12]; UK Biobank [46]; German National Cohort [2]; Rotterdam Scan Study [36]; SHIP [89]), such longitudinal analyses of RSFC changes in the aging brain are further enabled and help to disentangle the individual contributions of the different influencing factors on the older adult brain.

## References

[1] Andrews-Hanna JR, Snyder AZ, Vincent JL, Lustig C, Head D, Raichle ME, Buckner RL (2007). Disruption of large-scale brain systems in advanced aging. Neuron.

[2] Bamberg F, Kauczor HU, Weckbach S, Schlett CL, Forsting M, Ladd SC, Greiser KH, Weber MA, Schulz-Menger J, Niendorf T, Pischon T, Caspers S, Amunts K, Berger K, Bulow R, Hosten N, Hegenscheid K, Kroncke T, Linseisen J, Gunther M, Hirsch JG, Kohn A, Hendel T, Wichmann HE, Schmidt B, Jockel KH, Hoffmann W, Kaaks R, Reiser MF, Volzke H, German National Cohort MRISI (2015). Whole-body MR imaging in the German national cohort: rationale, design, and technical background. Radiology.

[3] Beckmann CF, DeLuca M, Devlin JT, Smith SM (2005). Investigations into resting-state connectivity using independent component analysis. Philos Trans R Soc Lond Ser B Biol Sci.

[4] Beckmann CF, Smith SM (2004). Probabilistic independent component analysis for functional magnetic resonance imaging. IEEE Trans Med Imaging.

[5] Betzel RF, Byrge L, He Y, Goni J, Zuo XN, Sporns O (2014). Changes in structural and functional connectivity among resting-state networks across the human lifespan. Neuroimage.

[6] Biswal B, Yetkin FZ, Haughton VM, Hyde JS (1995). Functional connectivity in the motor cortex of resting human brain using echo-planar MRI. Magn Reson Med.

[7] Biswal BB, Mennes M, Zuo X-N, Gohel S, Kelly C, Smith SM, Beckmann CF, Adelstein JS, Buckner RL, Colcombe S, Dogonowski A-M, Ernst M, Fair D, Hampson M, Hoptman MJ, Hyde JS, Kiviniemi VJ, Kötter R, Li S-J, Lin C-P, Lowe MJ, Mackay C, Madden DJ, Madsen KH, Margulies DS, Mayberg HS, McMahon K, Monk CS, Mostofsky SH, Nagel BJ, Pekar JJ, Peltier SJ, Petersen SE, Riedl V, Rombouts SARB, Rypma B, Schlaggar BL, Schmidt S, Seidler RD, Siegle GJ, Sorg C, Teng G-J, Veijola J, Villringer A, Walter M, Wang L, Weng X-C, Whitfield-Gabrieli S, Williamson P, Windischberger C, Zang Y-F, Zhang H-Y, Castellanos FX, Milham MP (2010). Toward discovery science of human brain function. Proc Natl Acad Sci U S A.

[8] Bittner N, Jockwitz C, Mühleisen TW, Hoffstaedter F, Eickhoff SB, Moebus S, Bayen UJ, Cichon S, Zilles K, Amunts K (2019). Combining lifestyle risks to disentangle brain structure and functional connectivity differences in older adults. Nat Commun.

[9] Boraxbekk CJ, Salami A, Wahlin A, Nyberg L (2016). Physical activity over a decade modifies age-related decline in perfusion, gray matter volume, and functional connectivity of the posterior default-mode network-a multimodal approach. Neuroimage.

[10] Button KS, Ioannidis JP, Mokrysz C, Nosek BA, Flint J, Robinson ES, Munafo MR (2013). Power failure: why small sample size undermines the reliability of neuroscience. Nat Rev Neurosci.

[11] Cabeza R (2002). Hemispheric asymmetry reduction in older adults: the HAROLD model. Psychol Aging.

[12] Caspers S, Moebus S, Lux S, Pundt N, Schütz H, Mühleisen TW, Gras V, Eickhoff SB, Romanzetti S, Stöcker T, Stirnberg R, Kirlangic ME, Minnerop M, Pieperhoff P, Mödder U, Das S, Evans AC, Jöckel K-H, Erbel R, Cichon S, Nöthen MM, Sturma D, Bauer A, Jon Shah N, Zilles K, Amunts K (2014). Studying variability in human brain aging in a population-based German cohort-rationale and design of 1000BRAINS. Front Aging Neurosci.

[13] Chan MY, Park DC, Savalia NK, Petersen SE, Wig GS (2014). Decreased segregation of brain systems across the healthy adult lifespan. Proc Natl Acad Sci U S A.

[14] Damoiseaux JS, Beckmann CF, Arigita EJ, Barkhof F, Scheltens P, Stam CJ, Smith SM, Rombouts SA (2008). Reduced resting-state brain activity in the "default network" in normal aging. Cereb Cortex.

[15] Davis SW, Dennis NA, Daselaar SM, Fleck MS, Cabeza R (2008). Que PASA? The posterior-anterior shift in aging. Cereb Cortex.

[16] Dickie DA, Job DE, Gonzalez DR, Shenkin SD, Ahearn TS, Murray AD, Wardlaw JM (2013). Variance in brain volume with advancing age: implications for defining the limits of normality. PLoS One.

[17] Farras-Permanyer L, Mancho-Fora N, Montala-Flaquer M, Bartres-Faz D, Vaque-Alcazar L, Pero-Cebollero M, Guardia-Olmos J (2019). Age-related changes in resting-state functional connectivity in older adults. Neural Regen Res.

[18] Ferreira LK, Busatto GF (2013). Resting-state functional connectivity in normal brain aging. Neurosci Biobehav Rev.

[19] Fjell AM, Sneve MH, Grydeland H, Storsve AB, Amlien IK, Yendiki A, Walhovd KB (2017). Relationship between structural and functional connectivity change across the adult lifespan: a longitudinal investigation. Hum Brain Mapp.

[20] Fjell AM, Sneve MH, Grydeland H, Storsve AB, de Lange AG, Amlien IK, Rogeberg OJ, Walhovd KB (2015). Functional connectivity change across multiple cortical networks relates to episodic memory changes in aging. Neurobiol Aging.

[21] Gargouri F, Kallel F, Delphine S, Ben Hamida A, Lehericy S, Valabregue R (2018). The influence of preprocessing steps on graph theory measures derived from resting state fMRI. Front Comput Neurosci.

[22] Geerligs L, Renken RJ, Saliasi E, Maurits NM, Lorist MM (2014). A brain wide study of age-related changes in functional connectivity. Cereb Cortex.

[23] Geerligs L, Rubinov M, Cam C, Henson RN (2015). State and trait components of functional connectivity: individual differences vary with mental state. J Neurosci.

[24] Goh JO (2011). Functional dedifferentiation and altered connectivity in older adults: neural accounts of cognitive aging. Aging Dis.

[25] Grady C, Sarraf S, Saverino C, Campbell K (2016). Age differences in the functional interactions among the default, frontoparietal control, and dorsal attention networks. Neurobiol Aging.

[26] Greicius MD, Krasnow B, Reiss AL, Menon V (2003). Functional connectivity in the resting brain: a network analysis of the default mode hypothesis. Proc Natl Acad Sci U S A.

[27] Griffanti L, Rolinski M, Szewczyk-Krolikowski K, Menke RA, Filippini N, Zamboni G, Jenkinson M, Hu MTM, Mackay CE (2016). Challenges in the reproducibility of clinical studies with resting state fMRI: an example in early Parkinson's disease. Neuroimage.

[28] Griffanti L, Salimi-Khorshidi G, Beckmann CF, Auerbach EJ, Douaud G, Sexton CE, Zsoldos E, Ebmeier KP, Filippini N, Mackay CE, Moeller S, Xu J, Yacoub E, Baselli G, Ugurbil K, Miller KL, Smith SM (2014). ICA-based artefact removal and accelerated fMRI acquisition for improved resting state network imaging. NeuroImage.

[29] Guerreiro R, Bras J (2015). The age factor in Alzheimer's disease. Genome Med.

[30] Harada CN, Natelson Love MC, Triebel KL (2013). Normal cognitive aging. Clin Geriatr Med.

[31] Hedden T, Gabrieli JD (2004). Insights into the ageing mind: a view from cognitive neuroscience. Nat Rev Neurosci.

[32] Heinrich A, Szostek A, Meyer P, Reinhard I, Gilles M, Paslakis G, Rauschenberg J, Gröbner J, Semmler W, Deuschle M (2014). Women are more strongly affected by dizziness in static magnetic fields of magnetic resonance imaging scanners. Neuroreport.

[33] Hirsiger S, Koppelmans V, Merillat S, Liem F, Erdeniz B, Seidler RD, Jancke L (2016). Structural and functional connectivity in healthy aging: associations for cognition and motor behavior. Hum Brain Mapp.

[34] Huang CC, Hsieh WJ, Lee PL, Peng LN, Liu LK, Lee WJ, Huang JK, Chen LK, Lin CP (2015). Age-related changes in resting-state networks of a large sample size of healthy elderly. CNS Neurosci Ther.

[35] Hughes C, Faskowitz J, Cassidy BS, Sporns O, Krendl AC (2020). Aging relates to a disproportionately weaker functional architecture of brain networks during rest and task states. Neuroimage.

[36] Ikram MA, Brusselle G, Ghanbari M, Goedegebure A, Ikram MK, Kavousi M, Kieboom BCT, Klaver CCW, de Knegt RJ, Luik AI, Nijsten TEC, Peeters RP, van Rooij FJA, Stricker BH, Uitterlinden AG, Vernooij MW, Voortman T (2020). Objectives, design and main findings until 2020 from the Rotterdam Study. Eur J Epidemiol.

[37] Jannusch K, Jockwitz C, Bidmon H-J, Moebus S, Amunts K, Caspers S (2017). A complex interplay of vitamin B1 and B6 metabolism with cognition, brain structure, and functional connectivity in older adults. Front Neurosci.

[38] Jockwitz C, Caspers S, Lux S, Eickhoff SB, Juetten K, Lenzen S, Moebus S, Pundt N, Reid A, Hoffstaedter F (2017). Influence of age and cognitive performance on resting-state brain networks of older adults in a population-based cohort. Cortex.

[39] Jockwitz C, Caspers S, Lux S, Jütten K, Schleicher A, Eickhoff SB, Amunts K, Zilles K (2017). Age-and function-related regional changes in cortical folding of the default mode network in older adults. Brain Struct Funct.

[40] Jones DT, Machulda MM, Vemuri P, McDade EM, Zeng G, Senjem ML, Gunter JL, Przybelski SA, Avula RT, Knopman DS, Boeve BF, Petersen RC, Jack CR (2011). Age-related changes in the default mode network are more advanced in Alzheimer disease. Neurology.

[41] Keulers EH, Stiers P, Nicolson NA, Jolles J (2015). The association between cortisol and the BOLD response in male adolescents undergoing fMRI. Brain Res.

[42] Laird AR, Eickhoff SB, Li K, Robin DA, Glahn DC, Fox PT (2009). Investigating the functional heterogeneity of the default mode network using coordinate-based meta-analytic modeling. J Neurosci.

[43] Lindbergh CA, Zhao Y, Lv J, Mewborn CM, Puente AN, Terry DP, Renzi-Hammond LM, Hammond BR, Liu T, Miller LS (2019). Intelligence moderates the relationship between age and inter-connectivity of resting state networks in older adults. Neurobiol Aging.

[44] Malagurski B, Liem F, Oschwald J, Merillat S, Jancke L (2020). Functional dedifferentiation of associative resting state networks in older adults - a longitudinal study. Neuroimage.

[45] Malagurski B, Liem F, Oschwald J, Mérillat S, Jäncke L (2020) Functional dedifferentiation of associative resting state networks in older adults–a longitudinal study. NeuroImage:116680 10.1016/j.neuroimage.2020.116680 32105885

[46] Miller KL, Alfaro-Almagro F, Bangerter NK, Thomas DL, Yacoub E, Xu J, Bartsch AJ, Jbabdi S, Sotiropoulos SN, Andersson JL, Griffanti L, Douaud G, Okell TW, Weale P, Dragonu I, Garratt S, Hudson S, Collins R, Jenkinson M, Matthews PM, Smith SM (2016). Multimodal population brain imaging in the UK Biobank prospective epidemiological study. Nat Neurosci.

[47] Mowinckel AM, Espeseth T, Westlye LT (2012). Network-specific effects of age and in-scanner subject motion: a resting-state fMRI study of 238 healthy adults. Neuroimage.

[48] Muller AM, Merillat S, Jancke L (2016). Small changes, but huge impact? The right anterior insula's loss of connection strength during the transition of old to very old age. Front Aging Neurosci.

[49] Muller AM, Mérillat S, Jäncke L (2016). Older but still fluent? Insights from the intrinsically active baseline configuration of the aging brain using a data driven graph-theoretical approach. Neuroimage.

[50] Mutschler I, Wieckhorst B, Meyer AH, Schweizer T, Klarhöfer M, Wilhelm FH, Seifritz E, Ball T (2014). Who gets afraid in the MRI-scanner? Neurogenetics of state-anxiety changes during an fMRI experiment. Neurosci Lett.

[51] Ng KK, Lo JC, Lim JKW, Chee MWL, Zhou J (2016). Reduced functional segregation between the default mode network and the executive control network in healthy older adults: a longitudinal study. Neuroimage.

[52] Nyberg L, Lovden M, Riklund K, Lindenberger U, Backman L (2012). Memory aging and brain maintenance. Trends Cogn Sci.

[53] Onoda K, Yamaguchi S (2013). Small-worldness and modularity of the resting-state functional brain network decrease with aging. Neurosci Lett.

[54] Oschwald J, Guye S, Liem F, Rast P, Willis S, Rocke C, Jancke L, Martin M, Merillat S (2019). Brain structure and cognitive ability in healthy aging: a review on longitudinal correlated change. Rev Neurosci.

[55] Park DC, Reuter-Lorenz P (2009). The adaptive brain: aging and neurocognitive scaffolding. Annu Rev Psychol.

[56] Perry A, Wen W, Kochan NA, Thalamuthu A, Sachdev PS, Breakspear M (2017). The independent influences of age and education on functional brain networks and cognition in healthy older adults. Hum Brain Mapp.

[57] Pietzuch M, King AE, Ward DD, Vickers JC (2019). The influence of genetic factors and cognitive reserve on structural and functional resting-state brain networks in aging and Alzheimer's disease. Front Aging Neurosci.

[58] Pillemer S, Holtzer R, Blumen HM (2017). Functional connectivity associated with social networks in older adults: a resting-state fMRI study. Soc Neurosci.

[59] Power JD, Cohen AL, Nelson SM, Wig GS, Barnes KA, Church JA, Vogel AC, Miezin FM, Schlaggar BL, Petersen SE, Laumann TO (2011). Functional network organization of the human brain. Neuron.

[60] Prehn K, Jumpertz von Schwartzenberg R, Mai K, Zeitz U, Witte AV, Hampel D, Szela AM, Fabian S, Grittner U, Spranger J, Floel A (2017). Caloric restriction in older adults-differential effects of weight loss and reduced weight on brain structure and function. Cereb Cortex.

[61] Pruim RH, Mennes M, van Rooij D, Llera A, Buitelaar JK, Beckmann CF (2015). ICA-AROMA: A robust ICA-based strategy for removing motion artifacts from fMRI data. Neuroimage.

[62] Raichle ME, MacLeod AM, Snyder AZ, Powers WJ, Gusnard DA, Shulman GL (2001). A default mode of brain function. Proc Natl Acad Sci U S A.

[63] Raz N, Lindenberger U, Rodrigue KM, Kennedy KM, Head D, Williamson A, Dahle C, Gerstorf D, Acker JD (2005). Regional brain changes in aging healthy adults: general trends, individual differences and modifiers. Cereb Cortex.

[64] Reuter-Lorenz P (2002). New visions of the aging mind and brain. Trends Cogn Sci.

[65] Reuter-Lorenz PA, Park DC (2014). How does it STAC up? Revisiting the scaffolding theory of aging and cognition. Neuropsychol Rev.

[66] Ritchie SJ, Cox SR, Shen X, Lombardo MV, Reus LM, Alloza C, Harris MA, Alderson HL, Hunter S, Neilson E, Liewald DCM, Auyeung B, Whalley HC, Lawrie SM, Gale CR, Bastin ME, McIntosh AM, Deary IJ (2018). Sex Differences in the adult human brain: evidence from 5216 UK Biobank participants. Cereb Cortex.

[67] Salami A, Pudas S, Nyberg L (2014). Elevated hippocampal resting-state connectivity underlies deficient neurocognitive function in aging. Proc Natl Acad Sci U S A.

[68] Salimi-Khorshidi G, Douaud G, Beckmann CF, Glasser MF, Griffanti L, Smith SM (2014). Automatic denoising of functional MRI data: combining independent component analysis and hierarchical fusion of classifiers. NeuroImage.

[69] Salthouse TA (2011). Neuroanatomical substrates of age-related cognitive decline. Psychol Bull.

[70] Schaefer A, Kong R, Gordon EM, Laumann TO, Zuo XN, Holmes AJ, Eickhoff SB, Yeo BTT (2018). Local-global parcellation of the human cerebral cortex from intrinsic functional connectivity MRI. Cereb Cortex.

[71] Schaie KW, Hofer SM (2001) Longitudinal studies in aging research.

[72] Shaw EE, Schultz AP, Sperling RA, Hedden T (2015). Functional connectivity in multiple cortical networks is associated with performance across cognitive domains in older adults. Brain Connect.

[73] Sheline YI, Raichle ME (2013). Resting state functional connectivity in preclinical Alzheimer’s disease. Biol Psychiatry.

[74] Siman-Tov T, Bosak N, Sprecher E, Paz R, Eran A, Aharon-Peretz J, Kahn I (2016). Early age-related functional connectivity decline in high-order cognitive networks. Front Aging Neurosci.

[75] Smith SM, Fox PT, Miller KL, Glahn DC, Fox PM, Mackay CE, Filippini N, Watkins KE, Toro R, Laird AR, Beckmann CF (2009). Correspondence of the brain's functional architecture during activation and rest. Proc Natl Acad Sci U S A.

[76] Smitha KA, Akhil Raja K, Arun KM, Rajesh PG, Thomas B, Kapilamoorthy TR, Kesavadas C (2017). Resting state fMRI: a review on methods in resting state connectivity analysis and resting state networks. Neuroradiol J.

[77] Song J, Birn RM, Boly M, Meier TB, Nair VA, Meyerand ME, Prabhakaran V (2014). Age-related reorganizational changes in modularity and functional connectivity of human brain networks. Brain Connect.

[78] Sporns O (2013). Network attributes for segregation and integration in the human brain. Curr Opin Neurobiol.

[79] Spreng RN, Stevens WD, Viviano JD, Schacter DL (2016). Attenuated anticorrelation between the default and dorsal attention networks with aging: evidence from task and rest. Neurobiol Aging.

[80] Stern Y (2009). Cognitive reserve. Neuropsychologia.

[81] Stern Y, Barulli D (2019). Cognitive reserve. Handb Clin Neurol.

[82] Stumme J, Jockwitz C, Hoffstaedter F, Amunts K, Caspers S (2020). Functional network reorganization in older adults: graph-theoretical analyses of age, cognition and sex. Neuroimage.

[83] Stumme J, Jockwitz C, Hoffstaedter F, Amunts K, Caspers S (2020) Functional network reorganization in older adults: graph-theoretical analyses of age, cognition and sex. NeuroImage:116756 10.1016/j.neuroimage.2020.116756 32201326

[84] Talukdar T, Zamroziewicz MK, Zwilling CE, Barbey AK (2019). Nutrient biomarkers shape individual differences in functional brain connectivity: evidence from omega-3 PUFAs. Hum Brain Mapp.

[85] Tomasi D, Volkow ND (2012). Aging and functional brain networks. Mol Psychiatry.

[86] Tsvetanov KA, Henson RN, Tyler LK, Razi A, Geerligs L, Ham TE, Rowe JB, Cambridge Centre for A, Neuroscience (2016). extrinsic and intrinsic brain network connectivity maintains cognition across the lifespan despite accelerated decay of regional brain activation. J Neurosci.

[87] Varangis E, Habeck CG, Razlighi QR, Stern Y (2019). The effect of aging on resting state connectivity of predefined networks in the brain. Front Aging Neurosci.

[88] Varikuti DP, Hoffstaedter F, Genon S, Schwender H, Reid AT, Eickhoff SB (2017). Resting-state test-retest reliability of a priori defined canonical networks over different preprocessing steps. Brain Struct Funct.

[89] Volzke H, Alte D, Schmidt CO, Radke D, Lorbeer R, Friedrich N, Aumann N, Lau K, Piontek M, Born G, Havemann C, Ittermann T, Schipf S, Haring R, Baumeister SE, Wallaschofski H, Nauck M, Frick S, Arnold A, Junger M, Mayerle J, Kraft M, Lerch MM, Dorr M, Reffelmann T, Empen K, Felix SB, Obst A, Koch B, Glaser S, Ewert R, Fietze I, Penzel T, Doren M, Rathmann W, Haerting J, Hannemann M, Ropcke J, Schminke U, Jurgens C, Tost F, Rettig R, Kors JA, Ungerer S, Hegenscheid K, Kuhn JP, Kuhn J, Hosten N, Puls R, Henke J, Gloger O, Teumer A, Homuth G, Volker U, Schwahn C, Holtfreter B, Polzer I, Kohlmann T, Grabe HJ, Rosskopf D, Kroemer HK, Kocher T, Biffar R, John U, Hoffmann W (2011). Cohort profile: the study of health in Pomerania. Int J Epidemiol.

[90] Walhovd KB, Westlye LT, Amlien I, Espeseth T, Reinvang I, Raz N, Agartz I, Salat DH, Greve DN, Fischl B, Dale AM, Fjell AM (2011). Consistent neuroanatomical age-related volume differences across multiple samples. Neurobiol Aging.

[91] Weis S, Patil KR, Hoffstaedter F, Nostro A, Yeo BTT, Eickhoff SB (2020). Sex classification by resting state brain connectivity. Cereb Cortex.

[92] Wu X, Li Q, Yu X, Chen K, Fleisher AS, Guo X, Zhang J, Reiman EM, Yao L, Li R (2016). A triple network connectivity study of large-scale brain systems in cognitively normal APOE4 carriers. Front Aging Neurosci.

[93] Yeo BT, Krienen FM, Sepulcre J, Sabuncu MR, Lashkari D, Hollinshead M, Roffman JL, Smoller JW, Zollei L, Polimeni JR, Fischl B, Liu H, Buckner RL (2011). The organization of the human cerebral cortex estimated by intrinsic functional connectivity. J Neurophysiol.

[94] Zhang HY, Chen WX, Jiao Y, Xu Y, Zhang XR, Wu JT (2014). Selective vulnerability related to aging in large-scale resting brain networks. PLoS One.

[95] Zonneveld HI, Pruim RH, Bos D, Vrooman HA, Muetzel RL, Hofman A, Rombouts SA, van der Lugt A, Niessen WJ, Ikram MA, Vernooij MW (2019). Patterns of functional connectivity in an aging population: the Rotterdam study. Neuroimage.

